# Familial dilated cardiomyopathy caused by a novel variant in the *Lamin A/C* gene: a case report

**DOI:** 10.1186/s12872-020-01695-8

**Published:** 2020-09-22

**Authors:** Jing Huang, Qing Wan, Yu Zou, Lijie Wang, Yesheng Pan

**Affiliations:** Department of Cardiology, Shanghai East Hospital, Shanghai Tongji University School of Medicine, No. 150 Jimo Road, Shanghai, 200120 China

## Abstract

**Background:**

Familial dilated cardiomyopathy (FDCM) is most commonly inherited as an autosomal dominant trait. The *Lamin A/C* (*LMNA*) gene variants have been identified to be associated with DCM, conductive system disorders, type 2 Emery-Dreifuss muscular dystrophy and several other disorders. Here, we reported a novel variant in the *LMNA* gene that might be related to FDCM.

**Case presentation:**

A 30-year-old young man was hospitalized for chest tightness, extreme fatigue, palpitation and impaired activity tolerance. He had clinical characteristics including cardiac dilatation, atrial tachyarrhythmia, severe conductive system disorders, and dyskinesia of both upper limbs and the neck. Genetic sequence analysis indicated that the patient carried a novel c.1325 T>C heterozygous *LMNA* gene variant. Catheter ablation and cardiac resynchronization therapy with pacing function (CRT-P) were performed to treat the arrhythmia.

**Conclusion:**

The variant c.1325 T>C is a novel variant in the *LMNA* gene that has not been previously reported. Young patients with DCM, conductive system disorders and skeletal myopathy should be alert to the possibility of *LMNA* gene variant. Cardiac resynchronization therapy (CRT) may be a reasonable choice for patient carrying a *LMNA* gene variant with third-degree atrioventricular block even if the left ventricular ejection fraction is preserved in order to prevent the deterioration of cardiac function caused by right ventricular pacing dependency.

## Background

Dilated cardiomyopathy (DCM) is a common primary cardiomyopathy that is characterized by the presence of ventricular dilatation and contractile dysfunction. Familial aggregation has been observed in some cases, called familial dilated cardiomyopathy (FDCM). Up to now, dozens of pathogenic gene variants have been identified, and among them, the *LMNA* gene is a major disease-causing gene [1]. Here, we reported a novel variant in the *LMNA* that might be related to FDCM and described its clinical features.

## Case report

**Fig. 1:**
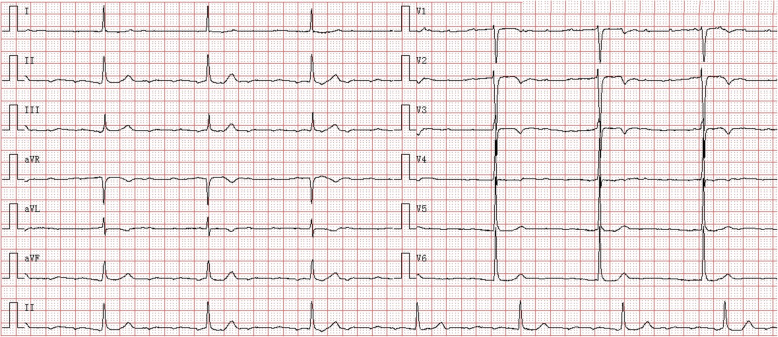
Baseline ECG showing rapid atrial arrhythmia (the atrial rate was approximately 180 beats per min) and third-degree atrioventricular block with junctional escape beat (the ventricular rate was approximately 43 beats per min, and the QRS duration was approximately 90 ms). ECG: 10 mm/mV, 25 mm/s

**Fig. 2:**
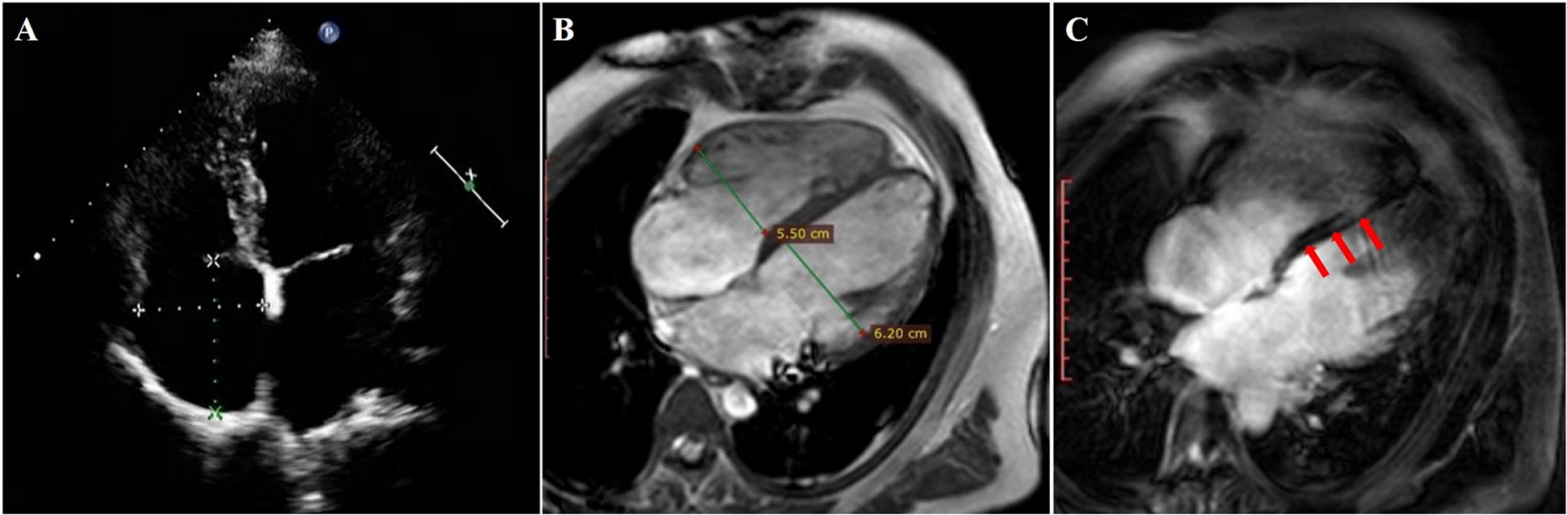
**a**. Transthoracic echocardiography (TTE) showing cardiac dilatation. The left atrial diameter was 44 mm, the left ventricular end diastolic diameter was 60 mm, and the left ventricular ejection fraction was 58%. **b**. Cardiac magnetic resonance imaging showing that the left ventricular end diastolic diameter was 62 mm and the right ventricular end diastolic diameter was 55 mm. **c**. Late midmyocardium gadolinium enhancement (red arrow)

A 30-year-old young man was hospitalized for chest tightness, extreme fatigue, palpitation and impaired activity tolerance. Laboratory examination revealed that myocardial enzymes were slightly elevated: high-sensitivity troponin T (cTnT) was 0.037 ng/ml (<0.014 ng/ ml), myoglobin (MYO) was 91.75 ng/ml (28–72 ng/ml), creatine kinase isoenzyme (CK-MB) was 7.86 ng/ml (0.1–4.94 ng/ml), and creatine kinase (CK) was 468 U/L (40–200 U/L). The N-terminal pro-brain natriuretic peptide (NT-proBNP) was 302 ng/L (<125 ng/L). Electrocardiogram (ECG) showed rapid atrial arrhythmia (atrial rate was approximately 180 beats per min) and sustained third-degree atrioventricular block with junctional escape beat (ventricular rate was approximately 43 beats per min, and the QRS duration was approximately 90 ms) (Fig. 1). There were no malignant ventricular arrhythmia events recorded by 24-h Holter monitoring. Transthoracic echocardiography (TTE) indicated cardiac dilatation (the left atrial diameter was 44 mm, and the left ventricular end diastolic diameter was 60 mm), and the left ventricular ejection fraction (LVEF) was preserved (58%) (Fig. 2a). Late midmyocardium gadolinium enhancement appeared in cardiac magnetic resonance perfusion imaging (Fig. 2b and c), which was consistent with the imaging findings of DCM.

**Fig. 3:**
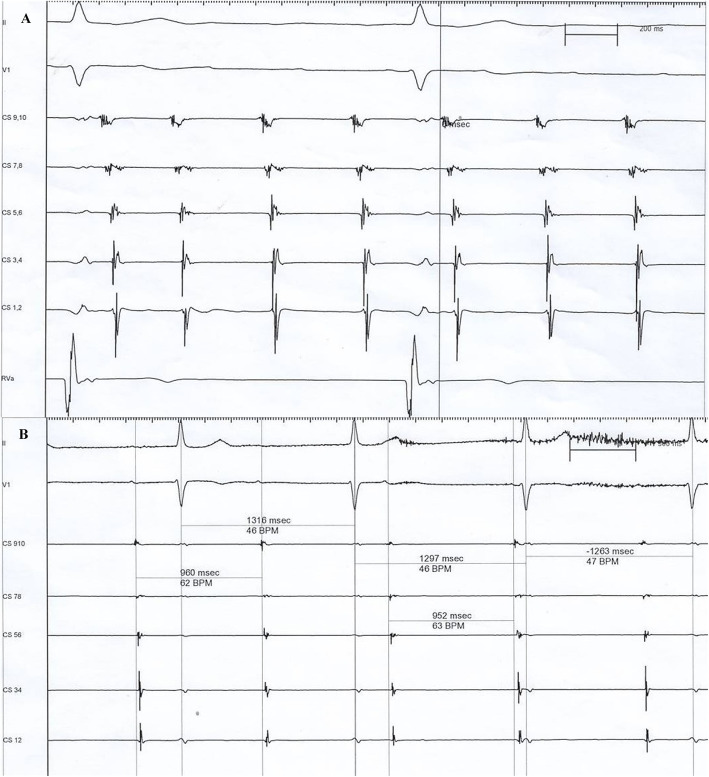
**a.** Coronary sinus electrogram showing that the coronary sinus ostium was activated the earliest. **b.** Intracardiac electrocardiogram indicating that the atrial rate was 63 beats per min, together with atrioventricular dissociation after liner ablation along the tricuspid valve isthmus

**Fig. 4:**
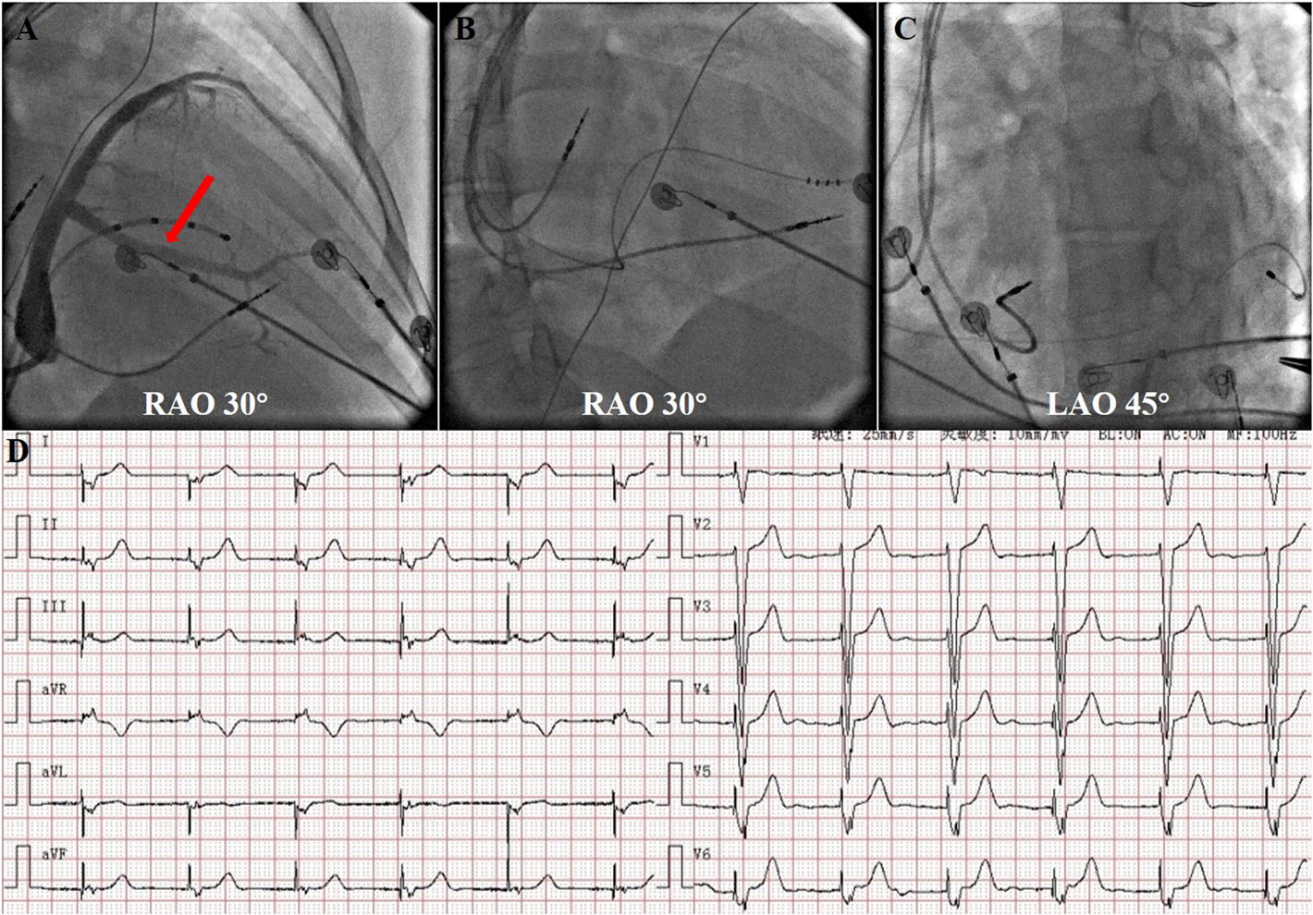
**a**. Coronary venography and target vein (red arrow) for left ventricular lead implantation. **b** and **c**. Fluoroscopic imaging after implantation of CRT-P (RAO: right anterior oblique; LAO: left anterior oblique). **d.** Postoperative ECG showing that the QRS duration was approximately 120 ms

To solve the problem of arrhythmia, an electrophysiologic study was performed. Atrial activation sequence was earliest in the coronary sinus ostium (Fig. 3a). During the process of atrial activation mapping, tachycardia was self-terminated and could no longer be induced by high-frequency atrial stimulation. As a matter of experience, it was considered more likely to be a cavotricuspid valve isthmus-dependent macro-reentry atrial tachycardia; thus, liner ablation along the cavotricuspid valve isthmus was performed. The normal atrial rate was only 40–63 beats per min together with atrioventricular dissociation (Fig. 3b). Permanent pacemaker implantation was extremely urgent. His-Bundle pacing (HBP) was first tried; unfortunately, the pacing lead was not only difficult to fix, but also, the capture threshold was higher than 3.0 V. As an alternative, biventricular pacing was successfully performed, and a triple-chamber pacemaker (model C2TR01, Medtronic Inc., Minneapolis, US) was implanted with a QRS duration of approximately 120 ms (Fig. 4).

**Fig. 5:**
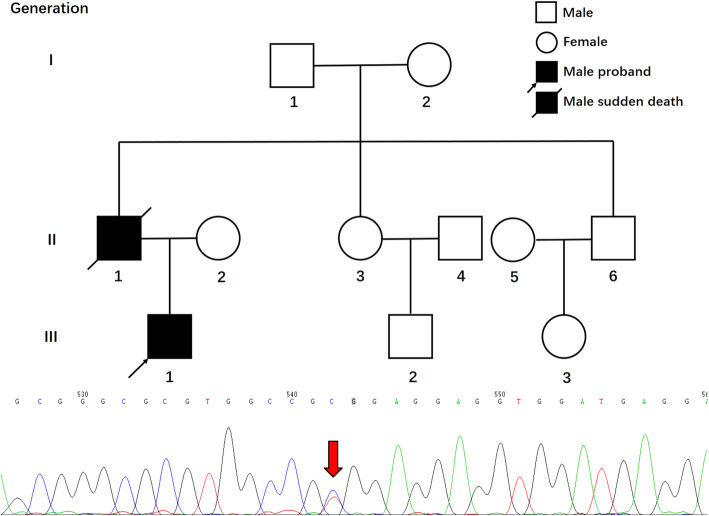
Pedigree of the family and chromatograms of the c. 1325 T>C (p. Val442Ala) heterozygous *LMNA* gene variant. Among other relatives, only the proband’s father’s brother (II-6) received genetic sequence analysis for this *LMNA* variant, but no variant was found

After operation, further physical examination found some specific manifestations, including mild muscle wasting of limbs, scoliosis, and disorders of upper-limb extension and neck flexion (the muscle strength was grade 2), which were also present in his father, who suffered from sudden death at the age of 42 years old. According to the above clinical features, inherited cardiomyopathy should be considered. Thus, genetic sequence analysis and pedigree investigation were performed, and a novel variant in the *LMNA* gene was found. The patient carried a c. 1325 T>C heterozygous LMNA gene variant resulting in a missense variant of amino acid 442 (p. Val442Ala) (Fig. 5).

After follow-up for more than 1 year, paroxysmal atrial fibrillation was observed with a burden of 35–40%, although no ventricular arrhythmia had occurred, and there was no further deterioration trend of the cardiac function. Oral drugs prescribed included candesartan, metoprolol and amiodarone.

## Discussion and conclusion

A pathogenic gene variant can be identified in approximately 40% of DCM patients [1, 2]. Once genes that encode cytoskeletal, sarcomere, and nuclear envelope proteins mutate, cardiomyopathy may occur and can have familial heredity. *LMNA* is the second major gene associated with a predominant cardiac phenotype, accounting for up to 5% of all cases of autosomal dominant DCM [3]. The *LMNA* gene encodes the Lamin A/C protein, variant of which may directly damage the stability of the nuclear membrane, often leading to conduction disorders and arrhythmia. More than forty gene locus variants of *LMNA* have been reported*.* Cardiac dilatation together with progressive lesion of the conductive system is more likely to be the initial or unique clinical manifestation, although *LMNA* variants are also associated with several disorders, such as type 2 Emery-Dreifuss muscular dystrophy, congenital muscular dystrophy, limb-girdle muscular dystrophy, Charcot-Marie-Tooth disease, and familial partial lipodystrophy type 2. DCM associated with *LMNA* variants results in a high sudden death rate, poor prognosis, age dependency (the average onset age is 33 years old), and high rates of major cardiac events [4]. Young DCM patients who have concomitant cardiac conduction disorders and supraventricular arrhythmia with or without skeletal myopathy and elevated CK level, especially those with symptoms of heart failure that appeared relatively later, should undergo *LMNA* gene testing.

The case we presented was consistent with the clinical features of *LMNA* gene-related FDCM. Further genetic sequence analysis was performed by a commercial entity (SinoPath Diagnosis, Beijing, China) using next-generation sequencing technology in March 2019. The expressed regions and adjacent intron regions of 376 genes associated with inherited cardiomyopathy and arrhythmia were captured. It was eventually found that the proband carried a heterozygous variant in *LMNA* gene (c. 1325 T>C) and amino acid missense (valine replaced by alanine in amino acid sequence 442) which has not been included in 1000 genome, ExAC, dbSNP, HGMD, Clinvar and OMIM database. Bioinformatics analysis software including SIFT, PolyPhen2 and Mutation_Taster all predicted that this variant was harmful. Genetic coordinates (GRCh37/hg19) is Chr1: 156106172 and the NM number is NM_170707.3. The This variant is autosomal dominant inheritance and regarded as variant of unknown significance according to ACMG classification. Pedigree investigation found that only the proband’s father had a history of skeletal myopathy and sudden death. Genetic variant was not found in his father’s brother, and other family members did not take part in the genetic sequence analysis because they live too far apart. Thus, the pathogenicity of this specific variant needs to be further ascertained through identifying additional patients, family segregation analysis, and animal studies. Additionally, DCM is acknowledged as a genetically heterogeneous disorder. According to the evidence presented in this study, other gene variants cannot be completely excluded.

Atrial tachyarrhythmia and atrioventricular block were the major problems of this patient. Catheter ablation of the cavotricuspid isthmus was empirically performed. However, it is unfortunate that atrial matrix mapping was not performed to determine whether there were some low-voltage areas in the right atrium. Permanent pacing was necessary due to third-degree atrioventricular block. It was important to note that right ventricular pacing dependency was foreseeable, which might have the potential risk of developing chronic pacing-related complications and cardiac function deterioration, especially for patients with DCM. Current guidelines also recommend that in patients with an LVEF between 36 to 50% and atrioventricular block, who have an indication for permanent pacing and are expected to require ventricular pacing over 40% of the time, CRT is preferred to right ventricular pacing to prevent heart failure [5]. Although our patient’s LVEF was over 50%, which was not in agreement with the recommendations of the current guidelines for physiological pacing techniques, such as CRT or HBP, considering that his heart was already enlarged and because the ventricular pacing dependency was foreseeable, right ventricular pacing alone could be harmful. For this reason, physiological pacing should first be considered. Due to the high capture threshold of HBP during the procedure, biventricular pacing was ultimately performed instead. Furthermore, cardiac conduction disorder caused by *LMNA* gene variant is deemed to progress continuously; hence, the pacing threshold of HBP will probably increase or result in loss of capture, which is inappropriate and insecure; thus, biventricular pacing may be more reasonable.

*LMNA* gene variant is also associated with an enhanced risk of life-threatening arrhythmic and sudden cardiac death [4]. Current clinical guidelines recommend permanent pacing with additional defibrillator capability if needed, and meaningful survival of greater than 1 year, as expected, is reasonable [5]. Previous research indicated that nonsustained ventricular tachycardia, LVEF<45% at the first clinical contact, male sex and non-missense variants were independent risk factors for malignant ventricular arrhythmias in *LMNA* variant carriers, and persons with at least 2 of these risk factors should be implanted with an implantable cardioverter-defibrillator (ICD) to prevent malignant ventricular arrhythmia events [6]. In our case, the neglect of important family history of his skeletal muscle disorders and the sudden death of his father, as well as the missed diagnosis of FDCM and carriage of an *LMNA* variant before the device implantation, resulting in us not assessing the indication of ICD to a large extent. Thus, a detailed history taking and physical examination are extremely important. However, our patient only had one risk factor (male sex), and no sustained ventricular tachycardia was observed. Thus, implanting a cardiac resynchronization therapy device without defibrillation function might be acceptable. Finally, for suspected FDCM combined with emergent pacing indications, whether to wait for the results of the genetic sequence analysis to determine the need for ICD implantation is open to question. The patient is being closely followed up, and if needed, the actual device will be upgraded with defibrillator capability at the proper time.

In conclusion, c. 1325 T>C (p. Val442Ala) is a novel variant in the *LMNA* gene that has not been previously reported. Young patients with DCM, conductive system disorders and skeletal myopathy should be alert to the possibility of *LMNA* gene variant. CRT may be a reasonable choice for patient carrying a LMNA gene variant with third-degree atrioventricular block even if the left ventricular ejection fraction is preserved in order to prevent the deterioration of cardiac function caused by right ventricular pacing dependency.

## Data Availability

Not applicable.

## Abbreviations

LMNA: Lamin A/C
FDCM: Familial dilated cardiomyopathy
DCM: Dilated cardiomyopathy
ECG: Electrocardiogram
TTE: Transthoracic echocardiography
LVEF: Left ventricular ejection fraction
HBP: His-bundle pacing
CRT: Cardiac resynchronization therapy
ICD: Implantable cardioverter defibrillator
